# Role of some epigenetic factors in DNA damage response pathway

**DOI:** 10.3934/genet.2017.1.69

**Published:** 2017-03-24

**Authors:** Mrinalini Tiwari, Suhel Parvez, Paban K Agrawala

**Affiliations:** 1 Department of Radiation Genetics and Epigenetics, Institute of Nuclear Medicine and Allied Sciences, Brig SK Mazumdar Road, Timarpur, Delhi 110054 India; 2 Department of Toxicology, Jamia Hamdard University, Hamdard Nagar, Delhi 110062 India

**Keywords:** DNA damage response, epigenetics, histone acetylation, radiomitigation

## Abstract

The current review gives a brief account of the DNA damage response pathway and involvement of various epigenetic mechanisms in DNA damage response pathway. The main focus is on histone modifications leading to structural alterations in chromatin since the compact chromatin structure poses a major limitation in the DNA repair process. Based on this hypothesis, our laboratory has also evaluated certain histone deacetylase inhibitors as potential radiomitigators and the same has been discussed in brief at the end of the review.

## 1. Introduction

Genomic integrity is challenged by various endogenous and exogenous DNA damaging agents continuously. Endogenous DNA damaging agents include many enzymatic conversions such as deamination, depurination, depyrimidination and many other replication errors, by-products of metabolic activities leading to generation of free radical species and increase in oxidative stress to the cells and are spontaneous in nature. These agents are more frequent and play a major role in the induction of genetic mutations in the cells. Among the various exogenous DNA damaging factors, ionizing radiations (IR) are the most important and are known to cause extensive damage to the genetic material. IR either directly interacts with the target molecule resulting in their ionization or indirectly with the molecules of cells including water to generate Reactive Oxygen Species (ROS) or free radicals which in turn then interact with different bio-molecules and cause damage to the DNA. DNA lesions can also be caused by non-ionizing radiations and various other chemical agents such as alkylating agents, benz(o)pyrene, aflatoxins and many electrophillic reactant metabolites, besides IR. Damage to the genetic material is primarily responsible for various types of double strand breaks (DSB) leading to mutations and other genomic instability, carcinogenesis and ageing. To preserve the genomic integrity, cells initiate a complex series of surveillance mechanism termed as DDR or DNA Damage Response collectively [1]. The inherited or acquired defects in DDR may result in severe diseased conditions like genetic instability, metabolic defects and carcinogenesis, etc [2]. The most fundamental features of DDR are detection of the DNA damage induced then signal its presence and then initiation of repair. DNA damage response involves two major series of events- cell cycle checkpoint activation and DNA repair pathways.

**Figure 1.**
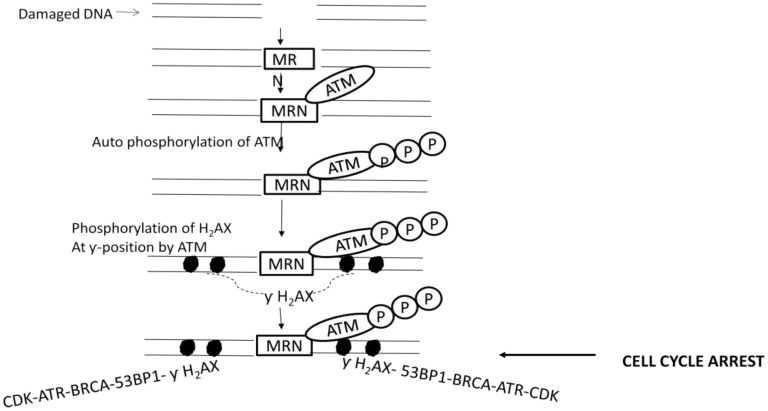
Schematic representation of DNA damage induced cell cycle checkpoint activation showing key epigenetic modifications.

## 2. Cell cycle checkpoint activation

In order to maintain the integrity of the genome and increase the survival of the organism, the pioneer step of DDR is activation of cell cycle checkpoint. Following checkpoint activation, cells may either undergo apoptotic cell death leading to their termination, or get silenced by senescence, or may survive after completion of the repair process which depends on the extent of damage and efficacy of repair machinery. Cell cycle checkpoints basically consist of a complex network of DNA damage sensors, signal transducers and many effector molecules or enzymes driven pathways. The checkpoints induced in response to DNA lesion recruit phosphoinositide 3-kinase related kinases (PIKKs) ATM, ATR and DNA-PK. These kinases actively participate in arresting the cells in their G1, S or G2 phase so that the repair machinery gets sufficient time to ensure efficient damage repair and restore genetic makeup [3]–[6]. The multifunctional Mre11-Rad50-Nbs1 (MRN) complex recognizes the DNA lesion in the initial phase of checkpoint activation [7]. The interaction of MRN complex to damaged site further mediates recruitment of ATM kinase through a protein-protein interaction between the C-terminal motif of NBS1 component of MRN complex and the HEAT repeats of ATM [8]. MRN-ATM interaction results in the activation of ATM kinase which leads to the autophosphorylation serine residues S1981 [9] and S367 and S1893 [10] of ATM. Activated ATM first of all phosphorylates the C-terminal tail of histone H2AX. H2AX phosphorylation provides the basis for a productive assembly of many other components of the DNA damage-modified chromatin. Other downstream targets of ATM are many signaling messengers such as checkpoint kinases (CHK1 and CHK2), 53BP1, BRCA1, ATR and cyclin-dependant kinases (CDK). These protein complexes take part in checkpoint initiation and signal propagation subsequently leading to termination of the response [11]. A schematic representation of cell cycle check point activation is made in Figure 1.

**Table 1.** DNA damages induced by various damaging agents and enzymes involved in the repair process.

Repair Mechanism	Type of DNA Damage	DNA Damaging Agents	Enzymes/Factors involved
Direct Repair	Non-enzymatic methylation of DNA by cellular S-adenosyl methionine	UV-induced	0-6 methyl guanine Methyl transferase
Base-excision repair	Oxidative lesion/Deaminated bases	ROS, X-rays. Alkylating agents Spontaneous	DNA glycosylases—UNG, OGG1, NEIL 1, MUTYH PARP1 AP endonuclease—APEX 1, APEX 2 DNA POL β XRCC 1 DNA Ligase 3
Nucleotide-excision repair	Broad DNA lesion distorting helix. DNA-protein adducts	UV induced, oxidative damage, environmental carcinogens	Global genomic RAD 23 B , TF II H, XPD Helicase, XPB ATPase, RPA, ERCC1, XPF II. Transcription coupled CSA, CSB, ERCC 1, XPF, XPG, XAB2, TREX 2
Homologous Recombination	Double strand DNA Break	Ionizing radiation ROS, Anti-tumor agent	ATM, NBN, BRCA1, BRCA 2 RAD 51, RAD 52 P53, WRN BLM, FANCB XRCC
NHEJ	Double strand DNA Break	Ionizing radiation ROS, Anti-tumor agent	KU70, KU 80 DNA-PK LIG 4 FEN1 XRCC 5

## 3. DNA repair pathways

DNA repair pathways allow cells to overcome the damage and survive with proper functioning of the genome although not absolute. There is also some redundancy in the functioning of the DNA repair pathways. If one pathway is disrupted, other pathway can be upregulated to compensate the repair process. Thus, the absence of any one pathway may increase the dependency on several other pathways. For example, (homologous recombination) HR repair deficient DNA lesions depend on error prone DNA repair process, (non-homologous end-joining) NHEJ [12]. The simplest and foremost mechanism of the repair of DNA lesions is direct reversal of DNA lesions by specialized enzymes such as Photolyase which reverses UV induced DNA damage or O-6-methyl guanine methyl transferase. Other more complex mechanisms are Base excision repair (BER), Nucleotide excision repair (NER), and non- homologous end joining (NHEJ). Table 1 summarizes various DNA damaging agents and the subsequent repair pathways initiated by the cells.

### 3.1. Base excision repair (BER)

BER process repairs DNA damages arising from oxidation, deamination or alkylation which normally cause little distortion in the DNA structure. The distortion may result in the formation of chemically altered base or single strand DNA break (SSB). The repair process is mediated by DNA glycosylase enzymes that cleave the N-glycosidic bond between the base and deoxyribose sugar thus removing the damaged base from DNA backbone and creating an abasic or AP site. The abasic (AP) site is incised by AP-endonucleases and the single nucleotide gap is filled-in by the BER-specific DNA polymerase β and finally sealed by the XRCC1/Ligase III complex. There are around 11 distinct mammalian DNA glycosylases of relatively overlapping specificities. The most interesting feature of BER is that the damaged base is easily identified and repaired without consumption of energy [13].

### 3.2. Nucleotide excision repair (NER)

NER removes a wide variety of structurally unrelated lesions including UV induced damage and many environmental carcinogens. NER pathway contains a complex series of almost 30 proteins which work in a highly versatile and coordinated manner to recognize and remove the DNA lesion. Genetic defect in one of the NER protein results in rare recessive hereditary diseases, xeroderma pigmentosum (XP), Cockayne syndrome (CS) and the photosensitive form of the brittle hair disorder trichothiodystrophy (TTD) [14]. Two modes of DNA repair are present in NER system. First is Global Genome NER (GG-NER), which localizes repair of DNA lesion over the entire genome and the second one is Transcription Coupled NER (TC-NER). TC-NER mediates the repair of transcription blocking DNA lesions which are present in the transcribed strand of DNA. The two pathways involve different proteins for damage sensing and then ultimately converge in the common subsequent steps. In GG-NER system, DNA lesion is recognized by two protein complexes: XPC/hHR23B [15] and UVDDB (DDB1 and DDB2/XPE) [16],[17]. Whereas in TC-NER, damage sensing is performed by the stalled RNA polymerase II, and the Cockayne syndrome factors A and B (CSA and CSB) play essential roles in TC-NER complex assembly [18]. After the recognition of the DNA lesions, NER/basal transcription factor TFIIH is recruited to the damaged site and bi-directional helicase of TFIIH opens the damaged DNA segment over a stretch of approximately 30 nucleotides. The unwound DNA is stabilized by Replication Protein A (RPA) and XPA and then the structure-specific endonucleases XPG and the ERCC1-XPF complex incise the damaged strand 3′ and 5′ with respect to the lesion, respectively. The resulting gap is then sealed by DNA ligase I and III [19].

### 3.3. Homologous recombination (HR)

HR is a homology-dependent repair pathway which requires the sister chromatid to seal the induced damage. It is a highly conserved process present in all life forms. HR is initiated by RAD51 protein which recognizes the break and binds to single strand DNA (ssDNA) coated with Replication protein A (RPA). RAD51 binds to ssDNA in cooperative manner forming RAD51-ssDNA pre-synaptic complex. Protein RPA has higher affinity and specificity for ssDNA than RAD51, hence there are certain mediator proteins which facilitates binding of RAD51 to RPA coated DNA. RAD55 and RAD57 are the two cofactors or mediators present in yeast whereas in humans there are five mediators, RAD51B, RAD51C, RAD51D, XRCC2 and XRCC3 [20]. These mediator molecules help in replacing RPA protein from ssDNA which is further occupied by RAD51 protein forming pre-synaptic complex. The synaptic complex catalyzes strand invasion mediating pairing of ssDNA with homologous dsDNA [21].

### 3.4. Non-homologous end joining (NHEJ)

It is the most commonly employed mechanism for DSB repair induced by ionizing radiations and free radicals. While both homologous recombination (HR) and NHEJ operate in the living system for DSB repair, NHEJ is more rapid and can repair the damage in HR deficient cells. It is an error prone DNA repair system which does not require sister chromatid for double strand break repair. It mostly operates in post mitotic cells and G1 phase of cell cycle. In NHEJ, the DSB is recognized by KU70/KU80 heterodimer which activates DNA PK and recruits it to the extreme end of the break. The two DNA PK of the extreme ends interacts with each other forming a synaptic complex which result in the autophosphorylation of the protein complex inducing conformational change. There is subsequent recruitment of nuclease artemis and MRE11/RAD50/NBS1 (MRN) protein complex. These protein complexes are involved in end processing and then ligation of the two ends is performed by XRCC4/LigaseIV complex [22].

## 4. Chromatin and its active role in DNA damage response

The nuclear DNA is structurally organized with the basic histone proteins into dynamic higher order chromatin structure which regulates and controls the gene expression. The nature and structural organization of chromatin proteins are very important contributors of cellular structure and function. The major cellular processes such as DNA replication, transcription and DNA repair require decondensation and subsequent restoration of structural integrity of the chromatin structure. Chromatin remodeling is the most important prerequisite for both the signaling and damage repair pathway. Since the DNA is packaged into chromatin, the structure of chromatin has great influence on efficacy of DNA Damage Response. Early in 1991, Smerdon [23] proposed a hypothetical model for DNA repair, “Access, Repair and Restore (ARR)” that for the first time proposed how the conserved DNA repair process operates in the living system. According to the ARR model, to initiate the DNA repair process, chromatin remodeling is required so that the damage recognition factors get “access” to the damaged site. Following damage recognition, DNA “repair” process starts and upon the completion of the repair process, chromatin “restores” its structure. The most important aspect determining the rate and efficiency of DNA repair is the chromatin remodeling events which include rearrangement of nucleosome structure resulting in formation of relaxed chromatin at DSB to allow repair machinery to access the regions surrounding DSB. Numerous histone modifications are involved in remodeling chromatin structure ultimately leading to relaxation of chromatin. These modifications include phosphorylation, methylation, acetylation, ubiquitinylation. The following sections describe each of the modifications in detail and its role in DDR.

### 4.1. Phosphorylation

Phoshorylation of histone is one of the most important requirements for the accumulation and binding of proteins involved in DDR. The discovery of the phosphorylation of H2A variant at S139 (γH2AX) is the earliest event in response to DNA damage [24]. H2AX phosphorylation is mediated by ATM which provides a docking platform for MDC protein through its BRCT domain. This results in the building up and activation of protein complexes (DDR factors) involved in the DNA damage response. The activated ATM of the DDR factors subsequently phosphorylates many substrates including CHK2 and KAP1 which spread the damage signal throughout the nucleus [25],[26] Although H2AX phosphorylation is predominantly detected in response to the DNA damage, it does not constitute the only primary signal required for the accumulation of repair factors to the site of DNA damage. It basically functions in concentrating protein factors in the vicinity of DNA lesions [27]. Studies also show that γH2AX does not spread uniformly throughout the chromatin and the size of the phosphorylated domain varies in different regions of the chromatin [28],[29]. γH2AX formation is important in the formation of ionizing radiation-induced foci (IRIF) after radiation injury although it has been found that even in the absence of γH2AX, the repair proteins are still recruited at the lesion site. This indicates that γH2AX-induced concentration of repair factors at sites of DNA damage does not play a crucial role in DNA repair [30]. Apart from H2AX phosphorylation, phosphorylation of two histone residues of H3, serine 10 and threonine 11 by CHK1 kinase triggers transcriptional activation during NER [31]. H2B is also phosphorylated at S14 however its function in DNA repair is not very clear.

### 4.2. Histone acetylation

Histone acetylation is an epigenetic modulation process of higher-order chromatin structure which is well known and extensively studied for increasing the accessibility of proteins to chromatin. It is one of the best characterized changes of chromatin organization that results in open conformation of even highly compact nucleosomes. Acetylation of histone is mostly associated with transcriptional activation, although its role in other chromosomal processes is relatively unexplored. The reversible acetylation and deacetylation of lysine residues of histones help to regulate protein-protein interactions and stabilize chromatin modifying enzymes at the site of DNA lesions. The primary targets of histone acetylation are H3 and H4 lysine residues. Acetylation neutralizes the basic charge of lysine residues thereby altering the interaction between adjacent histones and also between histone and DNA. This results in increased accessibility of DNA to different protein factors associated with chromosomal processes. In one of the earlier studies it was found that in response to UV irradiation, histones became hyperacetylated and DNA repair process was more efficient in hyperacetylated DNA [32]. Since then, several studies have implicated the role of chromatin acetylation in DNA repair. Acetylation and deacetylation of histone is highly controlled at the site of Double Strand Break (DSB) and many Histone Acetyl Transferases (HATs) and Histone Deacetylases (HDACs) are found to assemble and operate at DSB.

In UV induced DNA lesions, two major HATs are found to operate in increasing the acetylation of lysines in core histone octamer, KAT2A (GCN5) and EP300 (P300). In mammalian cells, KAT2A (GCN5) is involved in the acetylation of H3 and H4 histones at the site of DNA lesion. KAT2A (GCN5) makes part of the major protein complexes ATAC and SAGA, and has been shown to acetylate H3K9 in response to DNA damage. GCN5 mediated acetylation of H3 acts in conjugation with chromatin remodeling complex (SWI/SNF) to mediate efficient H2AX phosphorylation [33]. The bromodomain containing SWI/SNF ATPase BRG1 binds to acetylation histone and increases the accessibility of DNA lesion containing chromatin. Phosphorylated H2AX provides the docking site for KAT2A (GCN5) which increases the acetylation of H3 while acetylated histone recruit BRG1 further stimulating ATM mediated γH2AX formation. This γH2AX and acetylated H3 enriched microenvironment mediates recruitment of MDC1, BRCA1, 53BP1 forming effecter protein complex of DSB repair [34]. Depletion of KAT2A (GCN5) significantly affects the recruitment of repair proteins in the site of DNA lesion which ultimately impairs the repair mechanism.

Another histone acetyltransferase, P300/CBP HAT constitutively acetylates H2AX at K36 and H3 at K56 which is an important marker of chromatin site for DNA repair although the underlying mechanism of recognition is still not very clear [35]. Acetylation of H3 and H4 by P300/CBP significantly stimulates NHEJ as it has been found that DSB is greatly impaired in P300/CBP deficient cells [36]. CBP/p300 proteins were recruited to the site of DNA lesion and promote acetylation of H3K18, H4K5, H4K8, H4K12 and H4K16 which facilitates recruitment of KU70 and KU80, key proteins involved in NHEJ, implying acetylation a major contributor of DNA repair.

KAT5, a component of TIP60, is involved in transcriptional activation and repair of DNA lesion [37]. TIP60 consists of 14 distinct subunits and apart from acetylase, it has ATPase, Helicase and structural DNA binding activity. This broad range of activities suggest active role of TIP60 protein complex in DNA repair. It plays a very important role in acetylation of K5 of H2AX which is essential for ubiquitination of H2AX at K119 required for DNA damage signaling in response to ionizing radiation. Both ATM and NuA4 can complex with TIP60 for gaining HAT activity but seem to have different role in DNA damage repair. Acetylated H4 then further recruits protein complexes of DNA repair essential for homologous recombination [38],[39]. In response to ionizing radiations, H3K14 acetylation is also increased by the nucleosome binding protein, HMGN1. This acetylation indirectly regulates the activation of ATM kinase thus regulating DDR by inducing structural change in chromatin. Loss of HMGN1 or inhibition of its activity results in reduction of ionizing radiation dependent autophosphorylation of ATM and activation of several ATM targets [40].

Acetylation of H4K16 is another very important event regulating DDR. In response to DNA lesion, the steady state level of acetylation of H4K16 increases, through the enzyme KAT8 (MOF). It was found that in KAT8 (MOF) knockout mice, there was a global reduction in H4K16 acetylation and a defect in radiation induced DSB repair. KAT8 (MOF) is found to be associated with DNA- dependent protein kinase (DNA-PK) which is involved in NHEJ. Depletion of KAT8 (MOF) protein or its catalytic activity greatly impaired DSB repair by both homologous recombination and NHEJ. It was also observed that phosphorylation of DNA-PK by ATM in response to IR induced DNA damage was severely effected in MOF-deficient cells [40]. Both acetylation and deacetylation of lysines function in a reversible and highly dynamic manner to mediate DDR. For instance, following DSB induction, there is immediate deacetylation of H4K16 which is catalyzed by histone deacetylases, while the acetylation level increases in the later time point of DNA damage mediated by KAT8 (MOF). Histone deacetylases (HDAC1 and HDAC2) forms the subunits of nucleosome remodeling and NuRD complex which regulates efficient disassembly of repair factors, Artemis and KU which are essentially required for NHEJ of double strand break although the underlying mechanism is poorly understood. Similarly, acetylation of H3K56 is also reversible and highly dynamic at the site of DNA damage. Table 2 describes various enzymes leading to histone acetylation in response to DNA damage.

**Table 2.** Enzymes involved in histone acetylation and their role in DNA damage response (DDR).

HAT Enzyme	Part of Protein Complex	Acetylation site	Role
KAT2A(GCN5)	ATAC, SAGA	H3 & H4	- Acytalated H3 in conjugation with SWI/SNF mediates 𝓎-H2AX phosphorylation. - Recruitment of BRG 1 which stimulates ATM mediated γH2AX formation.
P300/CBP		H2AX at K36, H3 at K56 H4	- Stimulates NHEJ.
KAT5(TIP 60)	Nucleosome acetyl transferase of H4 (NuA4)	H2AX at K5 H4	- H2AX acetylation at K5 required for H2AX ubiquitination at K119 in DNA damage signalling. - Stimulates HR.
HMGN1 (HMGN1 dependent multiple HAT)		H3K14	- Activation of ATM kinase.
KAT8(MOF)		H4K16	- Stimulates phosphorylation of DNA-PK by ATM. - Activates HR and NHEJ.

### 4.3. Histone methylation

Another very abundant post transcriptional modification of histone involved in DNA damage response is histone methylation. Methylation occurs in either mono, di or trimethylated form in active lysine or arginine residues of histones. The type of methylation and residue involved in methylation serve as markers of either active or inactive chromatin. Methylation of lysine is relatively well studied as compared to arginine and is found to be associated with DDR. Methylation of lysine residues in histones regulates the recruitment and stabilization of gamma H2AX at the site of DNA lesion. Some of the important sites of histone methylation in response to DNA damage are H3K3, H3K9, H3K27, H3K36, H3K79, and H4K20. While methylation of H3K4, H3K36 and H3K79 are mostly associated with transcriptional activation, other methylated lysines such as H3K9, H3K27 and H4K20 are involved in transcriptional repression. The mono, di and trimethylated forms of lysine are regulated by lysine methyltransferases and lysine demethylases in DDR. Since methyl group is relatively small which does not significantly alter the basic charge of histone molecule, methylated lysines are basically involved in recruiting non -histone proteins at the site of DNA lesions. Repair of double strand break requires sister chromatid cohesion along the chromosomal arm which is provided by the cohesin complex. Cohesin complex is the key structural component of double strand break repair, single DSB induces a large domain of cohesin binding near the lesion. A different histone modification, methylation of H3, has also been established as being important for recruitment of cohesins to heterochromatin in S. pombe [41]–[43].

Methylation of H3K79 plays a major role in checkpoint function. Methylation of H3K79 is mediated by DOT1L and this methylation is required for DNA damage signaling to RAD53 [44]. H3K4 methylation by SETD1A also works in coordination with H3K79 methylation in controlling G1-S checkpoint. Studies show that mammalian 53BP1 fails to congress to the site of DNA lesion in absence of H3 methylation [45]. Cells deficient in SETD1A and H3K4 methylation show significant defects in repair by NHEJ and passes through replication stress [46]. Methylation of H3K4 takes place in mono, di or trimethylated forms. H3K4 me2 occurs in late S/G2 phase of cell cycle and regulates recruitment of 53BP1 protein involved in homologous recombination. Studies in budding yeast cells showed that H3K4me3 is required for proper response against DNA damaging agents and passage through S-phase of the cell cycle. Trimethylation of H3K4 by SETD1A is recognized as marker for newly synthesized DSB. The recruitment of SETD1A and is substrate at the site of DNA lesion is an essential requirement of maintaining genomic stability in yeast cells [47]. H3K4me3 is also required for binding of ING1 through its PHD finger domain. ING mediates cellular stress response such as DNA repair and apoptosis to prevent tumorigenesis [47].

Di and trimethylated H3K36 also participates in various DNA repair pathways involving HR and NHEJ. H3K36me3 (an active transcription marker) is recognized by the protein LEDGF (p75 splice variant) that promotes DNA DSB repair via homologous recombination by facilitating the recruitment of CtIP to DNA DSB and therefore promoting CtIP-dependent-DNA end-resection. H3K36me3 is also recognized by PHD domain of PHRF1 protein which binds to NBS1 and promotes NHEJ. H3K36me2 also binds to BRCT domain of NBS1 protein which helps in recruitment of MRN complex involved in NHEJ.

Another important methylation site involved in regulation of DNA repair is H3K9. The DNA damage induced H3K9me3 facilitates MRN-dependent recruitment and activation of KAT5 (TIP60) which ultimately acetylates and activates ATM protein kinase [48]. Trimethylated H3K9 is highly dynamic at euchromatin region. It primarily remains localized at heterochromatin regions in the absence of DNA damage. Following DSBs, a protein complex containing CAP-1, HP1 and SUV39H1 migrates to the site of DSB and catalyse trimethylation of H3K9 which in turn facilitates further recruitment of CAP-1, HP1 and SUV39H1 to the DSB site. This results in spreading of H3K9me3 to several kilobase segments and attachment of DDR factors [49]. Following DNA damage, there is global decrease in the level of H3K9 methylation which results in relaxation of chromatin. The global level of H3K9me2 decrease within 15 min of DNA damage which is recovered within 60 min whereas H3K9me3 level decrease within 5 to 15 min. This global decrease is important for chromatin relaxation in early and late time points of DNA repair.

H3K27 is also linked to local and gene specific transcriptional silencing following DNA damage. H3K27 is di and trimethylated by enzyme Polycomb repressive complex 2 (PRC 2) via its catalytic subunits EZH1 and EZH2. There is enrichment of EZH2 and H3K27me3 at the promoter site of DNA breakage. The elevated H3K27me3 contributes to PARP-dependent transcriptional silencing thus regulating DNA damage checkpoints.

Methylated H4K20 is recruited at 53BP1 site which is involved in the recruitment of DSB- responsive proteins, checkpoint signaling, DNA repair pathways and NHEJ following DNA damage. The exact role of H4K20-53BP1 interaction is not very well understood. However, studies showed that per-existence of H4K20me3 at damage site are critical for 53BP1 recruitment. Knockdown of enzyme responsible for H4K20 methylation showed delayed 53BP1 foci formation following radiation damage [50]. Different sites that are methylated in response to DNA damage are depicted in Table 3.

**Table 3.** Important sites of histone methylation and its role in DNA damage response (DDR).

Methylation site	Enzyme	Role
H3K79	DOT1L	DNA damage signalling to RAD 53
H3K4 (mono, di, tri)	SETD1A	- Recruitment of 53 BP1 - H3K4me2: Recruitment of 53BP1 - H3K4me3: Operates in early S-phase - Marker for newly synthesised double strand break. - Binding of ING1 through PHD domain
H3K36 (di, tri)	SET2, Nuclear receptor SET domain containing 1 (NSD 1)	- H3K36me2: binds to BRCT domain of NBS1 of MRN complex - Helps in recruitment of MRN complex - H3K36me3: Binds to NBS 1 of MRN complex through PHD domain
H3K9	CAP-1, HP1, SUV39H1	- Activation of TIP 60 - Relaxation of chromatin
H3K27 (di, tri)	Polycomb repressive complex 2 (PRC2)	- PARP dependent transcriptional silencing
H4K20	MMSET	-53BP1 foci formation to the site of DNA damage

### 4.4. Histone ubiquitination

Histone ubiquitination is a key regulatory modification in DNA damage response. It is observed that a series of ubiquitin-associated proteases and ubiquitin ligases are recruited directly to the sites of DNA lesions. Following DNA damage, there is ubiquitination of H2A and H2AX at K119 mediated by the RNF8 and RNF168 ubiquitin ligases, which are implicated in transcriptional silencing at DSBs. Ubiquitination of H2A is also critical for NER processing and ATR functioning. Ub-H2A further triggers recruitment of MDC1, BRCA1 and 53BP1 [51]. Besides RNF, BMI1 also mediates mono-ubiquitination of H2A since it was observed that depletion of BMI 1 resulted in impaired mono-ubiquitinated H2A but not poly-ubiquitinated H2A at DSBs that resulted in delayed recruitment of DDR factors. It is also found that BMI 1recruitment is dependent on gamma H2AX and RNF8, which suggests that BMI1 acts downstream to these protein factors [52]. Although these enzymes are critical for ubiquitination of histones at DSB sites, the exact mechanism of regulation of the overall process is not clearly understood. In one of the study it was found that H2A is ubiquitinated in the cytoplasm and after UV induced radiation injury, ub-H2A is recruited to the site of DSB following DNA repair [53].

In addition to ub-H2A, H2B ubiquitination mediated by the RNF20/RNF40 ubiquitin ligase complex is also found to play an important role in DNA damage response by both HR and NHEJ. Depletion of RNF20 resulted in lower level of P53 and 53BP1 expression suggesting its role in both DDR and tumorigenesis [54].

**Figure 2.**
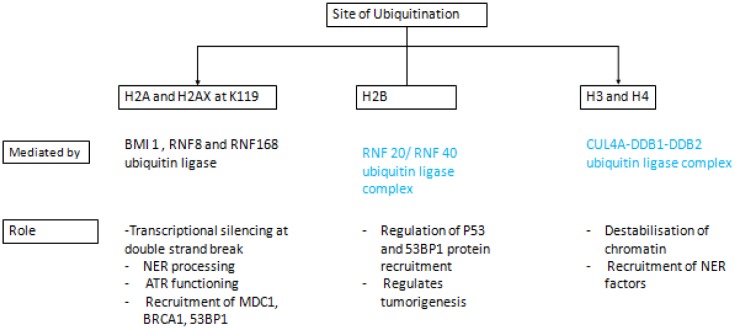
Important sites of histone ubiquitination and its role in DNA damage response (DDR).

H3 and H4 are also ubiquitinated in response to UV induced radiation injury mediated by protein complex CUL4A-DDB1-DDB2. Ubiquitination of H3 and H4 result in destabilization of chromatin and enhanced recruitment of NER factors to the site of DNA lesions [55]. Ubiquitination of various DDR proteins and responsible enzymes are shown in Figure 2.

## 5. Conclusion

Based on the available knowledge about the role of chromatin modification in response to DNA damage, an obvious connection between modulation of chromatin structure by histones and DNA damage response emerges. The dynamics of chromatin structure provides an excellent platform for the assembly of DNA damage associated factors and systematic recruitment and activation of protein complexes are the key factors determining the efficacy of DDR. Many new chromatin modulators have been identified in recent years that create a more accessible micro-environment suitable for the assembly of repair machineries. However, in order to obtain a clear understanding of the complex network of chromatin modifying factors and its dynamics, novel and more systematic approaches such as protein tagging, advanced confocal imaging, quantitative proteomics should be employed.

During past few years our laboratory has been engaged in evaluating some epigenetic modifiers, histone deacetylase inhibitors (HDACI) in particular, as mitigators of radiation injury [56]. This is based on the assumption that application of HDACI may facilitate all the downstream events of DNA metabolism, especially DNA repair, by leading to an open chromatin conformation and thus providing increased accessibility of the repair proteins/complexes to DNA damage sites induced by radiation exposure. The results obtained so far by our group in cell culture [57] and in a mice model system [58] in this direction are quite encouraging and interesting. However, the increased survival outcomes by HDACI post-irradiation administration to cells and mice may not solely be due to increased DNA repair only, and gene expression etc could play a significant role in the same. However, further experimental evidences are required to arrive at any conclusion.
